# A Bayesian framework to estimate diversification rates and their variation through time and space

**DOI:** 10.1186/1471-2148-11-311

**Published:** 2011-10-21

**Authors:** Daniele Silvestro, Jan Schnitzler, Georg Zizka

**Affiliations:** 1 Biodiversity and Climate Research Centre (BiK-F), Senckenberganlage 25, 60325 Frankfurt am Main, Germany; 2 Department of Botany and Molecular Evolution, Senckenberg Research Institute, Senckenberganlage 25, 60325 Frankfurt am Main, Germany; 3 Diversity and Evolution of Higher Plants, Institute of Ecology, Evolution and Diversity, Goethe University, Senckenberganlage 31, 60325 Frankfurt am Main, Germany

## Abstract

**Background:**

Patterns of species diversity are the result of speciation and extinction processes, and molecular phylogenetic data can provide valuable information to derive their variability through time and across clades. Bayesian Markov chain Monte Carlo methods offer a promising framework to incorporate phylogenetic uncertainty when estimating rates of diversification.

**Results:**

We introduce a new approach to estimate diversification rates in a Bayesian framework over a distribution of trees under various constant and variable rate birth-death and pure-birth models, and test it on simulated phylogenies. Furthermore, speciation and extinction rates and their posterior credibility intervals can be estimated while accounting for non-random taxon sampling. The framework is particularly suitable for hypothesis testing using Bayes factors, as we demonstrate analyzing dated phylogenies of *Chondrostoma *(Cyprinidae) and *Lupinus *(Fabaceae). In addition, we develop a model that extends the rate estimation to a meta-analysis framework in which different data sets are combined in a single analysis to detect general temporal and spatial trends in diversification.

**Conclusions:**

Our approach provides a flexible framework for the estimation of diversification parameters and hypothesis testing while simultaneously accounting for uncertainties in the divergence times and incomplete taxon sampling.

## Background

Patterns of species diversity have been shaped by both speciation and extinction throughout the history of life, and one of the key questions in evolutionary biology is to understand the temporal and spatial dynamics of these processes [1-6]. In addition to the fossil record, molecular phylogenetic data of extant lineages can provide valuable information on the process of diversification in form of branch length and the distribution of divergence times throughout the evolutionary history of a clade. Despite the omission of extinct lineages, it has been shown that differential patterns of speciation and extinction can leave a discernible signature on phylogenetic trees of extant taxa [7,8]. Methodological advances [9-14] as well as the growing number of well sampled, dated molecular phylogenies have generated considerable interest in unraveling the temporal dynamics of species diversification. Indeed, diversification rates have been assessed for a wide range of taxa from the tree of life to address questions concerning rapid radiations [15-18], mass extinction events [19], and differences among lineages [20,21] and geographic regions [11,22,23]. In particular, the identification of potential correlates of speciation and/or extinction rates, either extrinsic [e.g. climate or ecology; [24]] and/or intrinsic [e.g. key innovations; [25-28]], has received increased attention by relating rate shifts to external conditions or the evolution of species' traits.

Nee at al. [9] first applied the generalized birth-death process [29] to molecular phylogenies of extant lineages to extract information on the evolutionary process and proposed a likelihood approach to estimate both speciation and extinction rates (λ and μ, respectively). Given the need to distinguish different modes of diversification (e.g. deviations from constant rates), further approaches have been developed to incorporate rate variation through time [8,12,13,30] and across clades [14,31]. In addition, the original birth-death process was modified to correct rate estimates in case of incomplete taxon sampling [32-35].

While Bayesian Markov chain Monte Carlo (MCMC) methods are now commonly employed in phylogenetics to accommodate for uncertainties in model parameters, the temporal uncertainty of node age estimates is usually not taken into account when studying the dynamics of species diversification, resulting in an erroneous impression of precision. Here, we present a novel MCMC approach to estimate rates of speciation and extinction over the posterior distribution of trees generated in Bayesian molecular clock analyses. Several models of diversification have been implemented, including the constant rate birth-death and pure-birth processes modified to account for incomplete taxon sampling [33], a birth-death process with continuously varying rates [8], and a pure-birth process with rate shifts [12], for which the posterior distribution of λ and the temporal position of each rate shift are jointly estimated. Within the Bayesian framework, we describe a meta-analysis approach that aims at evaluating general patterns of species diversification across different taxonomic groups. In addition to the estimation of rate parameters, the approach presented here can also be used to distinguish between different modes of diversification, and test explicit hypotheses of rate variation through time and between clades using Bayes factors. We assess the power of the MCMC and of the Bayes factor test using simulated data sets, and demonstrate the application on empirical data sets: rate variation through time in the diversification of Mediterranean cyprinid genus *Chondrostoma*, geographic patterns in the radiation of the genus *Lupinus*, and a meta-analysis of four clades from the Cape flora of South Africa.

## Results

### Bayesian rate estimation across phylogenies

The birth-death process was implemented in a Markov chain Monte Carlo framework to estimate the parameters of species diversification (speciation and extinction rate) while accounting for phylogenetic uncertainty. Several modifications of the birth-death process originally described by Nee et al. [9] were implemented in the MCMC algorithm to describe different patterns of diversification and allow model selection and hypothesis testing. We included a modification of the birth-death process that accounts for incomplete taxon sampling based on Yang and Rannala [33] and Stadler [34] in which the fraction of the sampled species out of the total diversity (ρ) is used to correct the estimate of the diversification parameters. Although the missing species are assumed to be randomly distributed within the phylogeny, unlike in other models [e.g. [35]], we incorporate an option to assign different sampling fractions to predefined clades (see partitioned models below). In addition, Rabosky and Lovette's [8] SPVAR model was implemented to analyze the commonly observed pattern of "explosive-early" radiations, in which clades show an initial burst of diversification followed by a gradually declining speciation rate. Another model that accounts for rate variation through time is a pure-birth process in which a fixed number of shifts in diversification rate is assumed [12]. In contrast to the continuously varying birth-death process, the rate is assumed to vary only at specific times and otherwise remain constant. For a given number of rate shifts, the MCMC estimates their temporal position and the respective rates. Finally, our approach can be used to assign independent rates to predefined clades and is especially intended for hypothesis testing, complementing other approaches in which the rate constancy across-clades is relaxed [31], or in which the number and position of the rate shifts on the tree are estimated [14]. The data set is partitioned a priori by defining clades of interest, based for example on morphology or biogeography, and independent rates, models, and sampling proportions can be assigned to each clade.

### F-model: A meta-analysis approach

We develop a new method to investigate the strength and significance of general patterns of species diversification across different taxonomic groups through time or between clades in a meta-analysis framework. Within a collection of *N *data sets *d_1_, d_2_, ..., d_N _*(e.g. phylogenies of different taxonomic groups), each data set *d_i _*is partitioned *a priori *into two time frames or clades *d_i_*^(*p*) ^and *d_i_*^(*q*)^. The definition of these partitions can be based on criteria that are applicable to all phylogenies analyzed e.g. geologic events or geographic distribution. Their respective speciation rates λ_*i*_^(*p*) ^and λ_*i*_^(*q*) ^are described as a function of a multiplier *m_i _*and a parameter *F *so that:

(1a) $$\lambda_{i}^{\left(p\right)}=\frac{2m_{i}F}{1+F}$$

(1b) $$\lambda_{i}^{\left(q\right)}=\frac{2m_{i}}{1+F}$$

where *m_i _*= 1/2(λ_*i*_^(*p*) ^+ λ_*i*_^(*q*)^) represents a taxon specific mean rate that is assumed independent for each data set *d_i_*, and *F *= λ^(*p*)^/λ^(*q*) ^is constrained to be equal for all data sets and quantifies the overall magnitude of the rate difference between the two partitions. Based on these definitions, the rates are equal when *F *= 1, whereas λ^(*p*) ^> λ^(*q*) ^with *F *greater than 1, and λ^(*p*) ^< λ^(*q*) ^with *F *smaller than 1. We use MCMC sampling to obtain posterior estimates of the parameters *m_1_*, *m_2_*, ..., *m_N _*and *F *from the joint likelihood of all data sets *L_D_*

(2) $$L_{D}=\overset{N}{\underset{i=1}{\prod }}L\left[d_{i}^{\left(p\right)};\lambda_{i}^{\left(p\right)}\right]L\left[d_{i}^{\left(q\right)};\lambda_{i}^{\left(q\right)}\right]$$

Proposals for the parameters *m *are sampled from normal distributions centered on their current values, whereas new values of the *F *parameter are obtained from a log-normal distribution to achieve a symmetric proposal distribution in log(*F*). Reflection at the boundary was used to avoid proposals outside of the valid range (e.g. *m *≤ 0). Uniform priors are assigned to *m *in range [0, 10] and to log(*F*) in range [-2.3, 2.3], which corresponds to an *F *value in range [0.1, 10]. The clade-specific *F-*model can be extended to a birth-death process by assigning the parameter *m *to the mean net diversification (*r*) and introducing a second parameter *n *for the mean extinction fraction (*a*). Consequently, two parameters *F_r _*and *F_a _*are defined to measure the overall variation of *r *and *a *between clades of each data set. The significance of an overall rate difference across partitions is assessed via Bayes factor between a model in which *F *is allowed to vary and a model with constrained *F *= 1 (i.e. equal rates across partitions).

### Model selection using Bayes factor

Our analyses on simulated data sets show that the power of the Bayes factor test (BF) in finding the correct model is generally very high and not particularly affected by the model settings. Bayes factors were calculated between the model used to simulate each data set and a range of possible alternative models (Table 1) based on their respective marginal likelihoods (Additional file 1) obtained through thermodynamic integration [36-38]. Positive Bayes factors (Table 1) allow to correctly distinguish between diversification models in the majority of the simulations even in data sets with very low taxon sampling. Only when the extinction fraction is low (10%), the pure birth model obtains a slightly higher marginal likelihood than the birth-death. With variable rate pure-birth models, the number of rate shifts is correctly estimated when the magnitude of rate variation is moderate (fivefold) or higher. The effect of extinction and an increase in speciation rate in absence of extinction, both resulting in a similar pattern of increasing net diversification through time, can be distinguished with intermediate to high extinction fraction (> 50%) or a moderate (> twofold) rate increase. Furthermore, the power of Bayes factors in model selection improves with the size of the phylogeny (Table 1). For instance in case of a small (twofold) increase in the diversification rate, the correct model is found only on larger phylogenies (100 taxa).

**Table 1:** Bayes factors (BF) tests to distinguish different modes of diversification.

	Simulation settings				Bayes Factors (TDI)		
no. of tips (ρ)	λ	μ	BD	PB	PB2	PB3	PB4
50	0.5	0.05	**0**	-0.71	0.17		
100	0.5	0.05	**0**	-2.40	-1.77		
50	0.5	0.25	**0**	2.59	1.51		
100	0.5	0.25	**0**	3.66	-0.21		
50	0.5	0.45	**0**	24.98	3.57		
100	0.5	0.45	**0**	36.68	5.06		
50	0.5	0	1.05	**0**	1.18		
100	0.5	0	2.92	**0**	0.69		
100 (25%)	1	0	2.21	**0**			
200 (50%)	1	0	4.10	**0**			
300 (75%)	1	0	5.33	**0**			
400	1	0	6.37	**0**			
100 (25%)	1	0.9	**0**	36.65			
200 (50%)	1	0.9	**0**	68.30			
300 (75%)	1	0.9	**0**	98.18			
400	1	0.9	**0**	131.57			
50	0.1, 0.2	0	-0.59	1.74	**0**	1.76	
100	0.1, 0.2	0	0.44	6.78	**0**	0.88	
50	0.05, 0.25	0	4.64	23.73	**0**	1.22	
100	0.05, 0.25	0	5.79		**0**	0.73	
50	0.02, 0.16	0	13.94	40.60	**0**	0.69	
100	0.02, 0.16	0	29.53	90.60	**0**	0.93	
50	0.2, 0.1	0	4.02	0.54	**0**	1.47	
100	0.2, 0.1	0	11.57	5.57	**0**	1.53	
50	0.5, 0.1	0	23.22	18.52	**0**	1.49	
100	0.5, 0.1	0	58.15	51.49	**0**	0.81	
50	0.16, 0.02	0	23.92	19.51	**0**	0.85	
100	0.16, 0.02	0	56.32	49.53	**0**	0.60	
50	0.1, 0.2, 0.1	0	-0.55	-2.26	-1.28	**0**	2.04
100	0.1, 0.2, 0.1	0	4.81	0.35	0.69	**0**	1.52
50	0.1, 0.5, 0.1	0	12.93	12.56	8.26	**0**	0.67
100	0.1, 0.5, 0.1	0	45.38	40.22	21.47	**0**	-0.10
50	0.02, 0.16, 0.02	0	22.34	21.49	18.18	**0**	-1.11
100	0.02, 0.16, 0.02	0	63.95	64.01	54.67	**0**	-1.31

Bayes factors are calculated under birth-death (BD), pure-birth (PB), and pure-birth with rate shifts (PB2-PB4) based on thermodynamic integration. The BF values are calculated between the model applied in the simulation and the alternative models: Positive values support the true model.

### Rate estimation on simulated phylogenies

**Figure 1:**
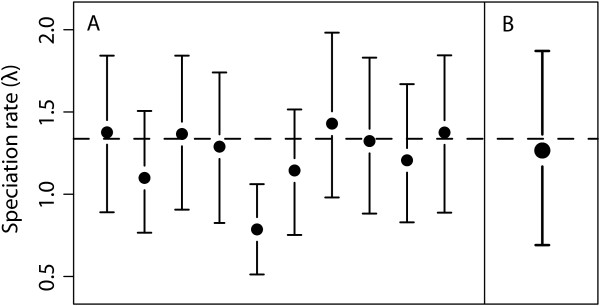
**Effect of accounting for phylogenetic uncertainty on rate estimates**. Comparison of the speciation rate estimated on 10 trees randomly sampled from the posterior distribution (A), and averaged over 100 trees (B). The dashed line indicates the maximum likelihood estimate based on the consensus tree. Error bars represent the 95% highest posterior density (HPD) interval of the rate estimates. Accounting for phylogenetic uncertainty results in an average increase of the width of the 95% HPD by 30% (B).

Analyses on simulated data sets indicate that the MCMC has a rather short burn-in phase and achieves a good chain mixing (measured as Effective Sample Size) with 110, 000 generations and a sampling frequency of 100. The posterior estimates of the speciation rate under the different models of diversification are found to be accurate with a relative error generally below 10% (Tables 2, 3, and 4). The relative error drops below 5% in data sets with 100 or more taxa, indicating that the size of the phylogeny has an impact on the accuracy of the parameter estimation. In addition the width of the rates' credibility intervals decreases with increasing size of the phylogeny: The HPDs are on average 25% narrower with 100 taxa compared to 50, and further reduce by another 50% with 400 tips (Additional file 2). In addition, about one third of the width of the 95% HPD is due to accounting for phylogenetic uncertainty (Figure 1).

**Table 2:** Estimates of speciation (λ) and extinction (μ) rates from simulated phylogenies.

Simulation settings			Estimates	
no. of tips (ρ)	λ	μ	**λ**_**MEAN **_**(rel. error)**	**μ**_**MEAN **_**(rel. error)**
50	0.5	0	0.47 (-0.07)	-
100	0.5	0	0.48 (-0.04)	-
50	0.5	0.05	0.57 (0.14)	0.23 (3.61)
100	0.5	0.05	0.55 (0.10)	0.18 (2.55)
50	0.5	0.25	0.49 (-0.03)	0.28 (0.11)
100	0.5	0.25	0.52 (0.03)	0.30 (0.20)
50	0.5	0.45	0.49 (-0.02)	0.43 (-0.04)
100	0.5	0.45	0.49 (-0.02)	0.44 (-0.03)
100 (25%)	1	0	1.09 (0.09)	-
200 (50%)	1	0	1.07 (0.07)	-
300 (75%)	1	0	1.06 (0.06)	-
400 (100%)	1	0	1.05 (0.05)	-
100 (25%)	1	0.9	0.99 (-0.02)	0.82 (-0.09)
200 (50%)	1	0.9	1.02 (0.02)	0.85 (-0.05)
300 (75%)	1	0.9	1.04 (0.03)	0.88 (-0.03)
400 (100%)	1	0.9	1.04 (0.04)	0.89 (-0.01)

Rates were inferred using the constant rate pure-birth or birth-death model, averaged over 100 phylogenies for each simulation. The taxon sampling (ρ) of incomplete phylogenies and relative errors are reported in parenthesis.

**Table 3:** Parameter estimation for the continuously varying birth-death process.

Simulation settings				Estimates		
no. of tips	λ	μ	*k*	**λ**_**MEAN **_**(rel. error)**	**μ**_**MEAN **_**(rel. error)**	***k***_**MEAN **_**(rel. error)**
50	1	0.1	0.25	1.91 (0.91)	0.50 (3.99)	0.23 (-0.08)
100	1	0.1	0.25	1.68 (0.68)	0.34 (2.38)	0.19 (-0.25)
50	5	0	0.95	3.72 (-0.26)	0.31 (-)	0.75 (-0.21)
100	5	0	0.95	5.22 (0.04)	0.33 (-)	0.97 (0.02)

Estimates of speciation (λ) and extinction (μ) rates and the *k *parameter are inferred using the variable rate birth-death model (SPVAR), averaged over 100 phylogenies. Relative errors are given in parenthesis.

**Table 4:** Rate estimates for the pure-birth process with rate shifts.

Simulation settings			Estimates	
no. of tips	λ	*s*	**λ**_**MEAN **_**(rel. error)**	***s***_**MODE**_
50	0.1, 0.2	5	0.11 (0.12), 0.24 (0.22)	4.86
100	0.1, 0.2	5	0.10, 0.22 (0.12)	4.81
50	0.05, 0.25	3.5	0.05, 0.26	3.59
100	0.05, 0.25	5	0.05 (0.07), 0.26	4.97
50	0.02, 0.16	5	0.02 (0.07), 0.17 (0.07)	4.95
100	0.02, 0.16	5	0.02, 0.16	5.05
50	0.2, 0.1	5	0.19, 0.12 (0.17)	5.09
100	0.2, 0.1	7	0.21, 0.11	6.97
50	0.5, 0.1	3	0.50, 0.11 (0.12)	3.06
100	0.5, 0.1	5	0.51, 0.10	5.04
50	0.16, 0.02	5	0.16, 0.03 (0.31)	5.05
100	0.16, 0.02	5	0.16, 0.02 (0.19)	5.01
50	0.1, 0.2, 0.1	3, 7	0.12 (0.22), 0.19 (-0.06), 0.12 (0.22)	3.01, 6.96
100	0.1, 0.2, 0.1	5, 10	0.10, 0.19 (-0.06), 0.11 (0.08)	4.88, 10.04
50	0.1, 0.5, 0.1	2, 4	0.11 (0.07), 0.32 (-0.36), 0.12 (0.21)	2.05, 3.95
100	0.1, 0.5, 0.1	2, 6	0.12 (0.18), 0.50, 0.11 (0.10)	1.94, 6.05
50	0.02, 0.16, 0.02	15, 20	0.03, 0.15, 0.03 (0.41)	15.49, 19.77
100	0.02, 0.16, 0.02	15, 20	0.02, 0.16, 0.02 (0.10)	15.48, 20.58

Speciation rates (λ) and temporal position of rate shifts (*s*) are inferred under the variable rate pure-birth model, averaged over 100 phylogenies for each simulation. The marginal rates are estimated as the mean of their posterior distributions, positions of rate shifts are estimated as the modal values of their posterior distributions. Relative errors are given in parenthesis if higher than 0.05.

**Figure 2:**
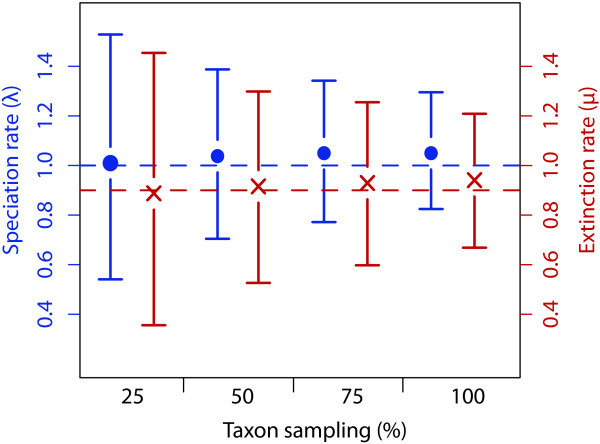
**Effect of taxon sampling on rate estimates**. Speciation (blue circles) and extinction (red crosses) rates estimated on simulated data sets of 400 taxa with a taxon sampling of 25%, 50%, 75%, and 100%, respectively. Phylogenetic trees were simulated with a speciation and extinction rate of 1 and 0.9, respectively (indicated by the dashed lines). Decreasing taxon sampling is correlated with an increase in the 95% highest posterior density (HPD) interval, whereas the accuracy of the rate estimate largely remains unaffected.

The constant rate birth-death model yields accurate posterior estimates of the speciation rate λ (Table 2) and efficient measures of the extinction rate are obtained when the extinction fraction is high (*a = *0.9). The accuracy of the estimate, however, decreases substantially when the extinction is low (*a = *0.1). This is likely due to the MCMC sampling, which is constrained by the fact that μ cannot become negative [32]. This results in a strongly skewed posterior distribution for which the mean is a poor estimator; a more accurate estimate is in this case provided by the mode. The 95% credibility interval of the posterior rates is always wide for μ (0 - 0.47 with μ = 0.05, *a *= 0.1, and 0.19 - 0.71 with μ = 0.45, *a *= 0.9; Additional file 2). While the sampling proportion does not significantly affect the accuracy in the estimation of λ and μ (Table 2), it has a strong impact on the width of the credibility intervals. The size of the 95% HPD increases by 20, 30, and 50 percent with ρ = 0.75, 0.5, and 0.25, respectively, compared to the complete data set (Figure 2).

The model with continuously decreasing diversification rates (SPVAR) yields accurate estimates of the parameter *k *(which determines the magnitude of the temporal decrease of the speciation rate; Table 3). On the other hand, the estimated initial speciation rate λ_0 _tends to be overestimated when *k *is small (with relative errors between 0.2 and 0.3), and underestimated for higher *k *values.

**Figure 3:**
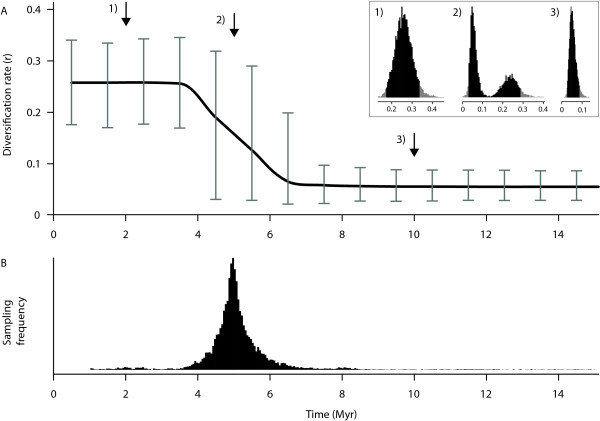
**Rates-through-time plot**. Diversification rates through time resulting from the analysis of 100 phylogenies simulated under a fivefold increase in diversification rates. The upper plot (A) shows the marginal rates for 1 Myr time categories (line) and the 95% highest posterior density (error bars). The x-axis represents time (Myr), and the y-axis is the average per-lineage diversification rate (spp/Myr). The insert displays three examples of marginal distributions of the diversification rate for three points along the phylogenies (indicated by arrows on the rates through time plot): 1) close to the tips (2 Mya), 2) at the point of rate shift (5 Mya), and 3) towards to root of the trees (10 Mya). Note the bimodal distribution of rates when a rat-shift is found (both the lower and higher rates are sampled). In the lower plot (B), the frequencies of a rate shift are proportional to the probability of a rate shift in that time frame.

Estimates of the speciation rates in data sets with rate-shifts were found to be accurate with a relative error on average lower than 0.1 (Table 4). The marginal rates estimated within 1 Myr time frames, reflect the rate variation through time (Figure 3). For time frames in which a rate shift occurs, the marginal rate is often represented by a bimodal distribution, which reflects the uncertainty on the temporal placement of the shift and results in an intermediate rate estimate with a wider 95% credibility interval (Figure 3A). This uncertainty is reflected in the frequency distribution of the rate shift in the posterior sample (Figure 3B). A highly accurate estimate of the time of rate shift is provided by the modal value of its sampling frequency, with relative errors lower than 0.05 (Table 4).

### Contrasting times of rate shift: Diversification of Mediterranean cyprinids

The use of a pure-birth process with rate shift and its implementation in hypothesis testing are illustrated in an analysis of the cyprinid genus *Chondrostoma *(Teleostei: Cyprinidae). A recently published molecular phylogeny of the genus [39] places the origin of the present lineages in the mid-Miocene around 15 Mya. Two alternative hypotheses on the diversification of *Chondrostoma *have been proposed, placing its radiation in the Mediterranean region either during the Messinian salinity crisis [40] or earlier in the Miocene [41]. The comparison of different models of diversification using Bayes factor tests led to the selection of a two-rate pure-birth process and estimated a fourfold decrease in speciation rates (dropping from an initial 0.441 to 0.108), and indicating a substantial slowdown during the Miocene. We used alternative two-rate pure-birth models to specifically test the fit of a rate shift during the Messinian [5.33 - 7.25 Mya; [40]] or earlier in the Miocene [7.25 - 23.03 Mya; [41]]. The rate shift was constrained in two separate analyses to lie within those periods, and the two models were compared by approximating a Bayes factor. Robalo et al. [42] favored the latter hypothesis based on their molecular clock analysis, although without specifically testing it in a statistical framework. Our analysis suggests that the Messinian Lago Mare phase had no particular effect on the radiation of the genus *Chondrostoma *(BF = 3.14), as a significant decrease in the speciation rate has to be placed before that period, thus supporting Robalo et al.'s conclusion.

### Clade-specific analysis: geographic patterns in the radiation of *Lupinus *(Fabaceae)

**Figure 4:**
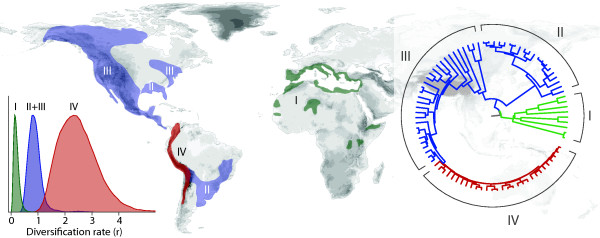
**Clade-specific analysis of the radiation of the genus *Lupinus***. Clades in the phylogenetic tree are labeled according to their distribution: I - Old World (Europe and North Africa), II - Eastern New World, III - Western North America and Central America, IV - Andean. Colors indicate the mean rates of diversification, with posterior rate estimates (relative densities) shown in the plot on the left (I - 0.20, II+III - 0.89, IV - 2.56). Note that the best model identified using Bayes Factors is a three-rate model in which clades II and III evolve at the same rate.

We demonstrate the application of models in which the rates can vary between predefined clades by analyzing the geographic patterns of diversification in *Lupinus *(Fabaceae) [16]. The phylogeny of the genus *Lupinus *shows a strong geographic structure which we used to define four partitions: I) an early diverging Old World clade, II) a group of eastern New World taxa, III) a clade occurring mainly in western North America and Central America, and IV) a clade including most of the Andean species (Figure 4). The latter displays a radiation from which around 80 species arose in the past 1.5 million years. While the model with equal rates among clades was strongly rejected in favor of variable rate models (Table 5), the highest marginal likelihood was assigned to a model with three different rates assigned to the clades I, II+III, and IV, respectively. The posterior distributions of the diversification rates are plotted as relative densities (Figure 4), showing a four-fold variation in speciation rate between the Old World *Lupinus *(λ_I _= 0.191) and the non-Andean New World lineages (λ_II+III _= 0.687). The Andean clade, as described by Hughes and Eastwood [16], represents an explosive radiation with a posterior rate estimate of λ_IV _= 2.510.

**Table 5:** Model comparison in *Lupinus*.

no. rates	partition settings	**L**_**M**_	BF
1	λ_I+II+III+IV_	-184.46	77.01
2	λ_I+II+III_, λ_IV_	-157.46	23.02
3	λ_I_, λ_II+III_, λ_IV_	-145.96	0
4	λ_I_, λ_II_, λ _III_, λ_IV_	-146.08	0.25

The marginal likelihoods (L_M_) of models with different partition settings are estimated via thermodynamic integration. Log Bayes factors (BF) are calculated by comparing the best fitting model (with 3 parameters) against all the others.

### A meta-analysis approach: Diversification of the Cape flora, South Africa

**Figure 5:**
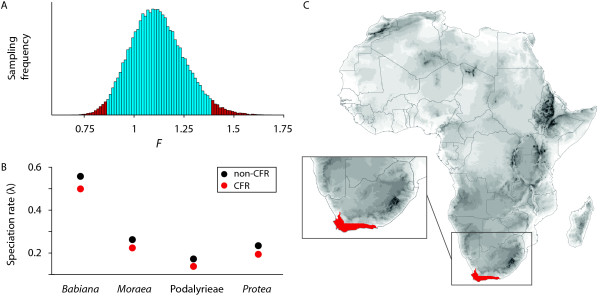
**Meta-analysis on plant diversification in the Cape Floristic Region (CFR)**. The map (C) shows the location of the CFR (highlighted in red) at the southwestern tip of the African continent. Rates of speciation (B) within the CFR (red) and in the rest of Africa (black) for each data set are found almost identical within and outside the CFR (*Babiana *0.5/0.556, *Moraea *0.259/0.288, Podalyrieae 0.15/0.167, and *Protea *0.195/0.218). The posterior density of the parameter *F *= 1.118 (95% HPD 0.86 - 1.39), (A) indicates overall similar rates in the two regions.

The high and unique plant diversity of the Cape Floristic Region (CFR) of South Africa is the result of an extraordinary contribution of lineages, that radiated extensively in the CFR [so called 'Cape floral clades'; [43]]. The high diversity and endemism suggest that Cape clades may have diversified at a faster rate within the CFR than elsewhere. Valente et al. [23] however recently showed that in the genus *Protea*, diversification rates in the Cape were, if anything, lower than in neighboring regions. To test for a general rate difference between Cape and non-Cape clades, we analyzed four data sets [23,44], all containing clades distributed within and outside the CFR, representing in total 537 plant species. The posterior rates estimated for the Cape/non-Cape clades of each individual data set are: *Babiana *0.500/0.556, *Moraea *0.259/0.288, Podalyrieae 0.150/0.167, and *Protea *0.195/0.218 (Figure 5B). The *F *parameter is estimated as 1.118 (95% HPD 0.86 - 1.39), indicating that diversification rates are overall slightly higher outside of the Cape (Figure 5A) region. However a Bayes factor of 4.78 between the constrained *F*-model with *F *= 1 (i.e. no rate difference between clades) and the unconstrained model suggests that this difference is not significant and that equal rates should be preferred. These results indicate an overall rate uniformity between Cape and non-Cape clades based on the four data sets analyzed, suggesting that the great diversity in the CFR might not be the result of a faster diversification process. It should be noted however that, as pointed out by Valente et al. [23], species ranges in these regions are vastly different, indicating that the key to understanding the Cape biodiversity hotspot instead lies in understanding why so many lineages have speciated and persisted in such a small area.

### Implementation

The method described has been implemented in a computer program called "BayesRate" (available at http://sourceforge.net/projects/bayesrate/ or from the authors) written in Python [45] (based on the Numpy [46] and Scipy [47] libraries) and R [48], integrating codes using the Python module rpy2 [49]. The thermodynamic integration supports multi-core computation by simultaneously running individual Markov chains on different processors. The log files can be examined with the program Tracer [50] to check for efficiency of the sampling (ESS), and convergence between independent runs.

## Discussion

We presented a new Bayesian approach, which provides a powerful tool to estimate rates of speciation and extinction on dated phylogenies based on the likelihood functions of the pure-birth, and birth-death processes while accounting for phylogenetic uncertainty and incomplete taxon sampling. On empirical data, our rate estimation requires a two steps analysis: 1) sampling a posterior distribution of dated phylogenies using a Bayesian molecular clock approach, and 2) estimating posterior diversification rates on these trees. Available programs such as BEAST [51] and mcmctree [52] apply in their relaxed molecular clock implementation different birth-death processes as priors on the node ages [53,54]. When applied to phylogenies obtained under these assumptions, our approach therefore requires that priors on the diversification parameters are specified twice, while ideally divergence times and diversification rates should be estimated jointly. The majority of the diversification processes considered here are however currently not implemented in these programs, and thus need to be estimated independently. Analyses on simulated data show that the default uniform priors on net diversification and extinction fraction in BEAST do not affect the subsequent rate estimates (relative rate variations lower than 3%; Additional file 3). Alternatively, researchers could choose to run molecular clock analyses in which the prior on the node ages is not based on a birth-death process, but modeled using uniform or Dirichlet distributions [55,56], as implemented in e.g. Multidivtime [57] and PhyloBayes [58].

The range of models implemented can be used to detect a number of different scenarios of rate variation, including specific events of rate increase or decrease, continuous rate variation through time, and clade-specific diversification rates, while simultaneously accounting for taxon sampling. In addition, the Bayesian framework extends the use of the birth-death models beyond the simple rate estimation allowing the comparison of alternative scenarios of diversification for hypothesis testing.

The Bayes factor test computed via thermodynamic integration has shown to represent a reliable and powerful approach to choose among different models of diversification. BF can be applied to compare non-nested models and does not require to be explicitly corrected for the number of model parameters. For these properties it is particularly suitable not only to select the best model in a Bayesian framework, but also to compare specific hypotheses. The power of Bayes factors to detect the correct number of rates predominantly depends on the magnitude of the rate shifts. Similarly, different birth-death and pure-birth processes can generate diversification patterns that might be difficult to distinguish [4,8]. For instance, an increase in the net diversification rate can be the result of an increased speciation rate in the absence of extinction or a high extinction rate in a constant rate birth-death process. Nevertheless, we found that the Bayes factors test has the power to discern between most of such scenarios.

Analyses on simulated data show that for both speciation and extinction rates the posterior estimates are accurate. However, the width of the 95% HPD intervals also highlights the sometimes considerable uncertainty in the parameter estimates, especially in case of small phylogenies. Our simulations have shown that this uncertainty is most pronounced with a low relative extinction rate, in which case extinction tends to be overestimated [see also [32]], and estimates have a wide 95% credibility interval. This corroborates previous studies that pointed out that the estimation of extinction rates from molecular phylogenies with reasonable degrees of confidence is very problematic [7,12,59,60]. It should be noted, however, that the wide credibility intervals reflect not only the uncertainties of the parameter estimation, but also the uncertainty of the data (i.e. the node ages). Estimates of the speciation rate on the other hand appear to be more robust. In contrast to Paradis [60], we find that the posterior estimate of λ has a small relative error, even under high relative extinction rates, suggesting that the accuracy in the estimates of λ might be decoupled from the relative rate of extinction. The pure-birth and birth-death models with taxon sampling are found to provide accurate rate estimates, although a poor sampling yields substantially wider 95% HPDs. Finally, the pure-birth process with rate-shifts tends to slightly underestimate the true variation of λ, as a consequence of accounting for the uncertainty of the time of rate shift.

The approach provides a very flexible framework for customized analyses and hypothesis testing such as predefined times of rate shift or constrained parameter values. We have shown with the diversification of *Chondrostoma*, that the pure-birth model with variable rates can be easily adapted to test for specific hypotheses, running the analysis on fixed time frames defined for example on the basis of geological events or climate changes. The implementation of clade-specific rate estimation further extends the range of options for hypothesis testing, and its application on the radiation of *Lupinus *showed that it can be used to identify differential rates of diversification between clades. In particular, the option to account for clade-specific sampling biases provides an important feature, as complete taxon sampling is often difficult to achieve, especially for species-rich groups.

Finally, with the *F*-model, we introduce a new approach to test hypotheses in a meta-analysis framework, and extend the focus from the taxon-specific rate of diversification to a parameter that might be linked to the difference between e.g. geologic periods or geographic regions. The current implementation allows to compare hypotheses with two rates, assigned to either fixed time frames or clades, while accounting for clade-specific taxon sampling. Because the *F *parameter is constant across data sets, we assume that the magnitude of the rate variation in time or between clades is equal among all data sets. While this certainly represents a simplification of the diversification process, the *F*-model allows the analyses of potentially many data sets, limiting the number of parameters, and yielding an estimation of general trends across different taxonomic groups. Moreover, even relatively small rate variations can be detected if supported by a sufficient number of data sets.

## Conclusions

In summary, the approach presented here shows that temporal dynamics of species diversification resulting from biologically relevant events such as key innovations or the impact of environmental change should best be studied in a Bayesian framework. The use of MCMC sampling provides an elegant way to estimate speciation and extinction rates while taking into account the often considerable uncertainty on divergence times. Furthermore, the model with taxon sampling represents an important step towards a more realistic estimation of the diversification parameters, where a non-random distribution of missing taxa can be incorporated with clade-specific sampling proportions. In addition to the models implemented in this study, recently developed modifications of the birth-death process [13,61] could also be integrated in the algorithm. With the possibility to run customized analyses specifically designed for hypothesis testing, this method provides a useful and flexible statistical framework to investigate diversification processes. A promising future development would be to relax the *F*-model to incorporate more than two rates, by assigning a specific multiplier to each time frame or group of clades.

## Methods

### Bayesian estimation of the diversification parameters

The likelihood of a birth-death process (BD) describing the speciation and extinction events of a dated phylogeny can be written as a function of the branching times ***x***, the number of extant species *s*, and the speciation and extinction rates λ and μ, respectively [9]:

(3) Lx;λ,μ=s-1!λ-μs-2 expλ-μ∑i=3sxi×1-μλs ∏i=2sexpλ-μxi-μλ-2

The function reduces to a pure-birth process (PB) in the absence of extinction (μ = 0).

We implemented this likelihood function in a Markov Chain Monte Carlo framework and applied the Metropolis-Hastings algorithm [62,63] to sample the posterior distribution of the birth-death model parameters. The algorithm is structured as follows:

1. Assign initial values to the model parameters (e.g. λ, μ)

2. Sample new *r*, *a *values (from which new λ, μ are obtained)

3. Accept or reject the proposal based on the acceptance probability

4. Repeat steps 2 and 3 many times

5. Repeat steps 1 to 4 over different trees sampled from their posterior distribution

6. Summarize the MCMC over all sampled trees by calculating mean and credibility interval for each parameter of interest

The MCMC iteration starts with random parameter values and successive proposals for λ and μ (step 2) are based on the sampling strategy described by Bokma [32], randomly drawing values of *r *= λ - μ (net diversification) and *a *= μ/λ (extinction fraction) from normal distributions centered on their current values. To avoid proposals lying outside of the valid interval (e.g. negative values) we use reflection at the boundary. The acceptance probability is proportional to the likelihood ratio, and uniform distributions are applied as flat priors on the rates. The MCMC is run over a distribution of trees, sampling λ and μ on each tree individually after a burnin phase (step 5) and the parameters of interest are summarized over all trees to account for the uncertainty on the node ages (step 6). The means of the posterior distributions of λ and μ are used as rate estimates and the respective credibility intervals are calculated as the 95% highest posterior density (HPD) intervals.

Assuming that a number of species are missing in a phylogeny, the missing lineages can be modeled as the result of an extinction event that occurs exactly at the present time [33]. Thus, when only a subset *s *of the total species *S *is included in the phylogeny, the likelihood of a set of branching times becomes a function of the proportion of sampled species ρ = *s*/*S*. This model assumes that taxon sampling is random with respect to the phylogeny. However, in case of a non-random sampling bias, individual clades in the phylogeny are represented to different extents. Thus, pure-birth and birth-death models were implemented in the MCMC framework with the possibility to assign a different sampling proportion (ρ) to each clade.

An approach to measure the variation of speciation and extinction rates through time has been introduced by Rabosky and Lovette [8], to model high initial rates of diversification followed by gradually declining net diversification rates. Their maximum likelihood method uses an exponential transformation of λ and μ through time with the introduction of two additional parameters, namely *k *and *z*, which specify the magnitude of λ decrease and μ increase, respectively:

(4a) λt=λ0 exp-kt

(4b) $$\mu \left(t\right)=\mu_{0}\left(1-\exp\left(-zt\right)\right)$$

where λ_0 _is the initial speciation rate, and μ_0 _the final extinction rate. A constant speciation rate is found with *k *= 0, whereas the extinction tends to be constant when *z *is very large. We implement Rabosky and Lovette's [8] SPVAR model (where speciation rates decrease through time while extinction rates remain constant) by applying a uniform prior in range [0, 10] for *k *and setting *z *to 10, 000. The parameters sampled by the algorithm are λ_0_, μ, and *k*.

The assumption of a pure-birth process (μ = 0) simplifies equation (3) as described by Kendall [29] and Nee et al. [9], and a likelihood-based approach has been described to detect shifts in diversification rates through time [12]. We implement this variable rate pure-birth model in which, given a number of rate shifts *n*, the estimated parameters are the temporal position of the shifts ***s ***= *s_1_, s_2_, ..., s_n_*, and the corresponding rates **λ **= λ_*1*_, λ_*2*_, *...*, λ_*n+1*_. Proposals for λ and *s *are sampled from normal distributions centered on their current values. The likelihood ratio is based on the product of the likelihoods of the branching times *x_i _*within each time frame delimited by *s_i-1_, s_i _*under the rate λ_*i*_:

(5) $$L\left(x;\lambda \right)=\overset{n}{\underset{i=1}{\prod }}L\left(x_{i};\lambda_{i}\right)$$

A uniform prior from 0 to the root age is assumed for the temporal position of the rate shift *s*. Because the temporal position of the rate-shift is not fixed but estimated through the MCMC sampling, we summarize the marginal rates as mean value and 95% credibility interval within predefined time frames (e.g. 1 Myr intervals) from the root age to the tips of the trees and use these estimates to draw rates-through-time plots (RTT). Thus, the marginal rates reflect the uncertainty on the time of rate shift. The sampling frequencies of the rate shifts through time are used to infer the temporal placement of the rate variation events. We identify the time of rate shift by finding the modal value of the frequency distribution of each parameter *s_i_*, representing the most frequently sampled value and approximating the *maximum-a-posteriori *estimate (MAP).

When testing for rate differences across predefined clades, the joint likelihood of all clades *C *is used to estimate the posterior distribution of the speciation and extinction rates (λ_c_, μ_c_) of each individual clade c. The parameters λ_c _and μ_c _can be constrained to be equal among clades (linked model) or estimated independently (unlinked model). A model comparison between linked and unlinked parameters is performed using Bayes factors (see below) to assess the significance of the rate difference between clades.

### Model selection: Bayes factors via thermodynamic integration

The fit of different models of diversification was assessed by comparing their respective marginal likelihoods, which are defined as the probability of the data *D *conditional on the model *M*, p(*D*|*M*). Alternative models can be compared using the Bayes factor test, which is defined as the ratio between their respective marginal likelihoods [64,65]. Calculating the marginal likelihood involves the integration of the probability of the data over the entire parameter space Θ:

(6) $$L_{M}=p\left(D|M\right)=\int_{\theta_{i}}p\left(D|\theta_{i},M\right)p\left(\theta_{i}|M\right)d\theta_{i}$$

Several approaches have been described to approximate *L_M_*. One simple approximation of *L_M _*is obtained as the harmonic mean of the likelihood values sampled via MCMC [65,66]. Although commonly used for model comparison in phylogenetics [e.g. [67,68]], the harmonic mean estimator has been found unstable, and thus often unreliable [37]. An alternative approach is thermodynamic integration (TDI) or path sampling [36-38], that has been shown to provide more accurate estimates of the marginal likelihood and has recently been applied in phylogenetics [37,69-71] and population genetics [72]. This method allows the exploration of regions of the parameters space with low likelihood by altering the acceptance ratio of the MCMC by a scaling factor *β*. The scaling factor ranges from 0 to 1 and is applied as an exponent to the likelihood function so that with *β *= 0 the MCMC samples from the prior distribution only, and with *β *= 1 the distribution of interest is sampled. The marginal likelihood *L_M _*is then obtained by integrating the likelihood expectations *E_β _*over all values of *β*:

(7) LM= ∫ 01Eβ lnpD∣θ,Mdβ

We discretize the integral by using a number *C *of scaling factors *β_0_, β_1_, ... β_C _*evenly spaced from 0 to 1, and estimating the respective log-likelihood expectations *U_βi _*as the mean of the MCMC sample. The discrete integral is then calculated applying Simpson's trapezoidal rule:

(8) $$L_{M}=\overset{\text{C}}{\underset{i=2}{\sum }}\frac{1}{2}\left(\beta_{i}-\beta_{i-1}\right)\left(U_{\beta_{i}}\text{ + }{\text{U}}_{\beta_{i}-1}\right)$$

The accuracy of this discrete approximation of (9) depends on the number of classes *C *that can represent a limiting factor since the computational time increases linearly with the number of categories. Beerli and Palczewski [72] showed that a highly accurate estimate of *L_M _*can be obtained with a small number of scaling factors when integrating analytically over the first interval [*β_0_, β_1_*] using Bézier cubic spline. This approach uses control points *P_1 _*and *P_2 _*based on the likelihood expectations at the first three scaling factors *U_0_, U_1_, U_2_*:

(9a) $$P_{1}=\left(\beta_{0},\frac{1}{5}U_{0}+\frac{4}{5}U_{1}\right)$$

(9b) $$P_{2}=\left(\beta_{0},\frac{\beta_{1}U_{2}-\beta_{2}U_{1}}{\beta_{1}-\beta_{2}}\right)$$

The four control points are used to define the cubic Bézier curve *B_P0, P1, P2, P3_*, with the first and the last points being

(10) $$P_{0}=\left(\beta_{0},U_{0}\right),P_{3}=\left(\beta_{1},U_{1}\right)$$

The integral of the marginal likelihood over the interval [*β_0_, β_1_*] is then calculated as

(11) $$\begin{array}{ccc} L_{M\left(\beta_{0},\beta_{1}\right)} & =\int_{\beta_{0}}^{\beta_{1}}B_{P_{0},P_{1},P_{2},P_{3}}d\beta & \\ & =\frac{1}{20}\left(\left(\beta_{1}-\beta_{0}\right)\left(U_{0}+3c_{y}^{\left(0\right)}+6c_{y}^{\left(1\right)}+10U_{1}\right)\right) \end{array}$$

We found that the shape of the Bézier spline described by Beerli and Palczewski [72] provided a good approximation of the curve obtained by applying many scaling factors under different models of diversification, and therefore adopted it in our computation of the marginal likelihood. After testing various numbers of scaling classes to calculate the discrete thermodynamic estimate of the marginal likelihood (not shown), six classes were found to be a good compromise between accuracy of the result and computational time. Once the log marginal likelihoods *L_M _*were obtained via TDI, the log Bayes factor (BF) between pairs of models *M_0 _*and *M_1 _*was computed as *BF_01 _*= 2(*M_1 _*- *M_0_*) and its interpretation based on the values suggested by Kass and Raftery [65]. Thus *BF_01 _*greater than 2 represent positive evidence for model *M_1_*, and greater than 6 provide strong evidence. To assess the power of Bayes factor in discerning between different modes of diversification, we analyzed several simulated data set under birth-death models and pure-birth assuming one to three rate shifts with a special focus on processes that generate similar patterns (e.g. birth-death and pure-birth with rate increase).

### Statistical evaluation

To test the performance of our method, we analyzed simulated phylogenies generated under a range of models using the R-package TreeSim [34,73]. A total of 38 data sets of 100 phylogenies (with 50, 100, or 400 tips) were simulated under different models of diversification (Additional file 4). We simulated constant rate birth-death models with extinction fractions ranging from low to very high (0.1, 0.5, and 0.9), and different taxon sampling proportions (25%, 50%, 75%, and 100%). Pure-birth processes were simulated with either constant rates, or including shifts in diversification rates (one or two shifts) under small (twofold), moderate (fivefold), and large (eightfold) rate variations, respectively (Additional file 4). Trees (50 and 100 tips) with approximately continuously decreasing speciation rates were obtained by imposing nine equally spaced rate shifts, under two different diversification scenarios where speciation rates follow an exponential decrease (λ_0 _= 1, μ = 0.1, and *k *= 0.25; λ_0 _= 5, μ = 0, and *k *= 0.95). Because of the limitations of the SPVAR [8] model under variable or high extinction rates [74,75], we assumed absent or very low and constant extinction. As these simulations only approximate continuously decreasing rates, we report the parameter estimates under the SPVAR model, but do not perform model comparisons via Bayes factors.

To assess the accuracy of the rate estimates, we calculated the relative errors [cf. [12]] as (*r*_est _- *r*_true_)/*r_true_*, where *r*_est _is the estimated rate of speciation or extinction and *r*_true _is the true value. A positive relative error indicates overestimation of the parameter, whereas a negative value indicates its underestimation. For the pure-birth model with rate variation, the marginal diversification rates through time were calculated for time categories of 1 million years, and their relative errors were calculated in relation to the true values between shift points. The modal values of the posterior distribution of the shift points were compared against the true shift times and their relative error was calculated as (*t*_est _- *t*_true_)/*T*, where *t*_est _is the estimated time of rate shift, *t*_true _is its true value, and *T *is the average root node age of the analyzed trees.

To address the impact of estimating rates on a single tree compared to analyzing a distribution of trees, we used a tree topology simulated in Phyl-o-Gen [76] under the birth-death process (100 tips; r = 1; *a *= 0.9) to simulate nucleotide sequences (3978 bp, HKY+I+Γ) using the program SeqGen [77]. Phylogenetic trees were then reconstructed in BEAST [v.1.6.1; [51]]. For comparison, we also inferred the maximum likelihood estimates of λ and μ on the consensus tree (Figure 3) through a birth-death optimization as implemented in LASER [78].

To empirically assess the potential impact of specifying priors on the birth-death parameters in both the molecular clock analysis and the subsequent rate estimation, additional simulations were performed. We generated trees in Phyl-o-Gen (50 tips; extinction fraction *a *= 0, 0.5, and 0.9) on which nucleotide sequences (5000 bp, HKY+I+Γ) were simulated using the program SeqGen [77]. Dated phylogenies were reconstructed in BEAST using the default uniform priors on the birth-death parameters and constraining the root node to the age of the initial tree. The posterior rates were then estimated using our MCMC approach on both the initial tree (used to simulate the alignment) and the distribution of trees obtained from BEAST. The rate estimates were compared by calculating their variation in terms of relative error (Additional file 3).

### Analyses on the case studies

The phylogenetic relationships of the genus *Chondrostoma *were reconstructed using mitochondrial cytochrome *b *and nuclear ß-actin gene sequences for all currently recognized taxa [39]. Phylogenetic trees and divergence times were reconstructed using BEAST [v.1.6.1; [51]] and assuming the GTR+I+Γ model of sequence evolution. A speciation model following a Yule process was selected as the tree prior, with an uncorrelated lognormal (UCLN) model for the rate variation among branches. Secondary calibration points were used, following Gante et al. [39], constraining nodes to a normal prior: the crown node of *Chondrostoma *was constrained with a mean of 15.1 Mya (central 95% range 12.5 - 17.6 Mya). The split between *C. olisiponensis *and its sister clade was constrained with a mean of 10.1 Mya (central 95% range 7.7 - 12.4 Mya). The analysis was run for 15 million generations, sampling states every 2, 000 generations. The adequacy of the sampling was assessed with Tracer [50] using the Effective Sample Size diagnostic (Additional file 2). We evaluated the temporal patterns of diversification using all diversification models implemented in our approach applied on a random sample of 100 trees from the molecular clock analysis.

We reconstructed the phylogeny and divergence times of the genus *Lupinus *based on a combined alignment of ITS and LEGCYC1. Following Hughes and Eastwood [16], the two markers were partitioned and analyzed under GTR+Γ and GTR+I models, respectively. A relaxed molecular clock analysis was carried out using BEAST, assuming an uncorrelated lognormal clock model, running 30 million MCMC generations. A normal distribution with a mean of 16.01 Mya and a standard deviation of 2.6 for the stem node of *Lupinus *was set as calibration point. We carried out the diversification analyses on a random sample of 100 trees obtained from a relaxed molecular clock analysis, applying a pure-birth process after model selection. The estimation of the diversification rates was performed assuming clade-specific taxon sampling (ρ_I _= 0.77, ρ_II _= 0.55, ρ_III _= 0.18, ρ_IV _= 0.40) under different models in which the rates were linked or unlinked among clades.

The meta-analysis on the Cape Floristic Region was based on four dated phylogenies of different Cape clades [23,44]. The analyses using the *F-*model were performed on a random set of 100 trees sampled from the posterior distribution of each data set. All clades have on average only a small proportion of missing taxa, which was accounted for by means of clade-specific sampling fractions. Each data set was split into Cape and non-Cape clades based on their main geographic distribution, high degrees of endemism - particularly within the CFR - allowed a simple assignment of clades (Figure 5C). The meta-analysis was performed implementing pure-birth models, which were favored over a birth-death process with a Bayes factor value of 3.46.

## Authors' contributions

DS, JS, and GZ designed the study. DS and JS developed the Bayesian framework and the models. DS wrote the Python code, JS designed and performed the simulations and wrote the R scripts. DS and JS wrote the paper, all authors read and approved the final version of the manuscript.

## Supplementary Material

Additional file 1 **Marginal Likelihoods for different models of diversification**. Marginal Likelihoods for simulated data sets calculated under birth-death (BD), pure-birth (PB), and pure-birth with rate shift (PB2-PB4) models based on thermodynamic integration. Click here for file

Additional file 2 **Posterior rate estimates**. Parameter estimates, 95% credibility intervals and ESS values for all data sets simulated. All simulations settings are provided in Additional file 4. Click here for file

Additional file 3 **Effect of sequential estimation of divergence times and diversification rates**. The potential impact of specifying priors on the birth-death parameters in both the molecular clock analysis and the subsequent rate estimation is assessed through generating a starting tree, simulating a molecular alignment on it, and run BEAST analyses on the alignment. The rate are then estimated on both the starting tree and the BEAST posterior trees, and compared. Click here for file

Additional file 4 **List of the simulation settings**. Simulations were obtained from birth-death (BD), pure-birth (PB), pure-birth with rate shift (PB2-PB4), and (approximately) continuously decreasing speciation rates (SPVAR). The parameters included are speciation rates (λ), extinction rate (μ), time of rate shift (*s*), sampling fraction (ρ), and the shape parameter of the exponential transformation of λ through time (*k*). The continuously decreasing rates (SPVAR model) were approximated by imposing nine equally spaced rate shifts where speciation rates follow an exponential decrease. The value of λ reported for the SPVAR model represents the initial speciation rate (λ_0_). Click here for file
